# A Comprehensive Analysis Revealing FBXW9 as a Potential Prognostic and Immunological Biomarker in Breast Cancer

**DOI:** 10.3390/ijms24065262

**Published:** 2023-03-09

**Authors:** Shiyi Yu, Zhengyan Liang, Zhehao Fan, Binjie Cao, Ning Wang, Rui Wu, Haibo Sun

**Affiliations:** 1 Institute of Translational Medicine, Medical College, Yangzhou University, Yangzhou 225001, China; 007585@yzu.edu.cn (S.Y.); 18202730560@163.com (Z.L.); fzhehao@126.com (Z.F.); yzucbj@163.com (B.C.); wn18435166884@163.com (N.W.); wr15004421751@163.com (R.W.); 2 Jiangsu Key Laboratory of Experimental & Translational Non-Coding RNA Research Yangzhou, Yangzhou 225001, China

**Keywords:** FBXWs, FBXW9, ubiquitin, immunology, pan-cancer

## Abstract

The WD40 repeat-containing F-box proteins (FBXWs) family belongs to three major classes of F-box proteins. Consistent with the function of other F-box proteins, FBXWs are E3 ubiquitin ligases to mediate protease-dependent protein degradation. However, the roles of several FBXWs remain elusive. In the present study, via integrative analysis of transcriptome profiles from The Cancer Genome Atlas (TCGA) datasets, we found that FBXW9 was upregulated in the majority of cancer types, including breast cancer. FBXW expression was correlated with the prognosis of patients with various types of cancers, especially for FBXW4, 5, 9, and 10. Moreover, FBXWs were associated with infiltration of immune cells, and expression of FBXW9 was associated with poor prognosis of patients receiving anti-PD1 therapy. We predicted several substrates of FBXW9, and TP53 was the hub gene in the list. Downregulation of FBXW9 increased the expression of p21, a target of TP53, in breast cancer cells. FBXW9 was also strongly correlated with cancer cell stemness, and genes correlated with FBXW9 were associated with several MYC activities according to gene enrichment analysis in breast cancer. Cell-based assays showed that silencing of FBXW9 inhibited cell proliferation and cell cycle progression in breast cancer cells. Our study highlights the potential role of FBXW9 as a biomarker and promising target for patients with breast cancer.

## 1. Introduction

Although advances have been achieved in understanding their mechanisms, cancers remain a major threat to humans and a serious public health problem worldwide [1]. Owing to the public easily accessed database, pan-cancer analysis has been performed on many proteins and non-coding RNAs [2,3], which could provide valuable information for cancer studies given that hallmarks are shared in cancers of various origins [4].

Ubiquitination is a crucial biological process for protein turnover [5,6]. This process is achieved via the cooperation of the E1 enzyme, E2 enzyme, and E3 ligase [7,8]. F-box proteins are the most well-characterized E3 ligases among hundreds [9]. Generally, F-box proteins specifically bind to phosphorylated substrates, leading to proteolytic ubiquitination (Lysine 48, Lysine 11) or non-proteolytic ubiquitination (Lysine 63) [10]. F-box proteins can be divided into three classes, namely FBXL (22 members), FBXO (37 members), and FBXW (10 members) [11]. For FBXWs, these proteins contain a WD-40 domain, which is pivotal for its recognition of substrates [12]. Several members of FBXWs have been recently identified as oncogenes or tumor suppressors according to cell background [13,14,15]. For example, FBXW7 mediated the ubiquitination of BGN to regulate peritoneal metastasis of gastric cancer [13], while the elevation of FBXW4 was observed in acute myeloid leukemia and its expression was associated with poor prognosis of patients [14]. FBXW1 was involved in the maintenance of cancer stem cells in glioblastoma via targeting GLI2 [15]. However, the roles and functions of most members of FBXWs have not been extensively examined in cancers.

In the current study, we performed a comprehensive bioinformatic analysis of FBXWs in several large cohorts covering most cancer types. Our analysis reveals the expression pattern and potential roles of FBXWs in the prognosis, stemness, and immune infiltration of cancers. The potential pathways and targets of FBXW9 were also studied. We also performed cell-based assays to validate the pro-cancer function of FBXW9 in breast cancer cells.

## 2. Results

### 2.1. Analysis of mRNA Levels of FBXWs in Tumors of Various Cancer Types and Normal Tissues

**Figure 1:**
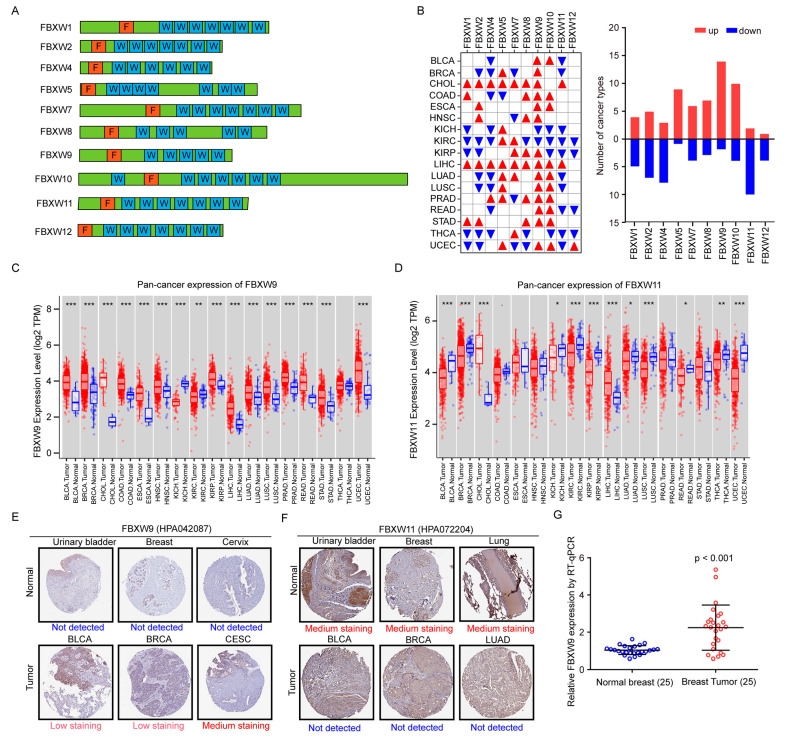
Analysis of FBXW expression in pan-cancer tissues and normal tissues. (**A**) Schematics of the architecture of FBXWs. F: F-box domain. W: WD40 domain. (**B**) The expression of mRNA levels of FBXWs was analyzed in 17 cancer types and normal tissues from the TCGA database. Red triangle: overexpressed. Blue triangle: downregulated. The number of cancer types with differentially expressed FBXWs was counted and summarized. (**C**) The expression of FBXW9 in 17 cancer types compared with normal tissues is presented in box plots. (**D**) The expression of FBXW11 in 17 cancer types compared with normal tissues is presented in box plots. (**E**) Representative images of immunohistochemistry (IHC) data of FBXW9 staining in tumors and normal tissues from the Human Protein Atlas (HPA) database. (**F**) Representative images of IHC data of FBXW11 staining in tumors and normal tissues from the Human Protein Atlas (HPA) database. (**G**) RT-qPCR detection of FBXW9 mRNA expression in 25 pairs of tumors and normal tissues from patients diagnosed with breast cancer. *, *p* < 0.05; **, *p* < 0.01; ***, *p* < 0.001.

All members of FBXWs contain an F-box domain and multiple WD40 domains (Figure 1A). The mRNA levels of all 10 members of FBXWs were retrieved from TCGA datasets including tumors of 17 cancer types and normal tissues. The expression of most FBXWs was positively correlated with the others in all cancers (Figure S1). All FBXWs were dysregulated in at least one cancer type (Figure 1B). Of all FBXW members, FBXW9 was the most frequently upregulated across 17 cancer types (14/17), while FBXW11 was the most frequently downregulated gene in cancers (10/17) (Figure 1B). Noticeably, the most significant upregulation of FBXW9 was observed in invasive breast carcinoma (BRCA) (*p* = 9.10 × 10^−19^), followed by colon adenocarcinoma (COAD) (*p* = 2.30 × 10^−18^), bladder urothelial carcinoma (BLCA) (*p* = 7.60 × 10^−10^), and 11 other cancer types (*p* < 0.001 for all) (Figure 1C). In contrast, downregulation of FBXW11 was observed in BLCA, BRCA, kidney chromophobe (KICH), and 7 other cancer types (Figure 1D). To examine the protein expression of FBXW9 in tumors and normal tissues, we analyzed immunohistochemistry (IHC) data in the Human Protein Atlas database. FBXW9 protein was not detected in normal tissues (urinary bladder, breast, and cervix). In contrast, low or medium staining of FBXW9 protein was observed in tumors of corresponding types (BLCA, BRCA, and cervical squamous cell carcinoma and endocervical adenocarcinoma (CESC)) (Figure 1E). In contrast, IHC staining of FBXW11 showed that FBXW11 was expressed in the urinary bladder, breast, and lung, but was not expressed in BLCA, BRCA, and lung adenocarcinoma (LUAD) (Figure 1F). We further evaluated FBXW9 mRNA expression in our collected breast tumors, which also showed upregulation of FBXW9 mRNA in tumors compared with normal tissues (Figure 1G).

### 2.2. Analysis of the Association between mRNA Levels of FBXWs and the Prognosis of Patients with Cancer

**Figure 2:**
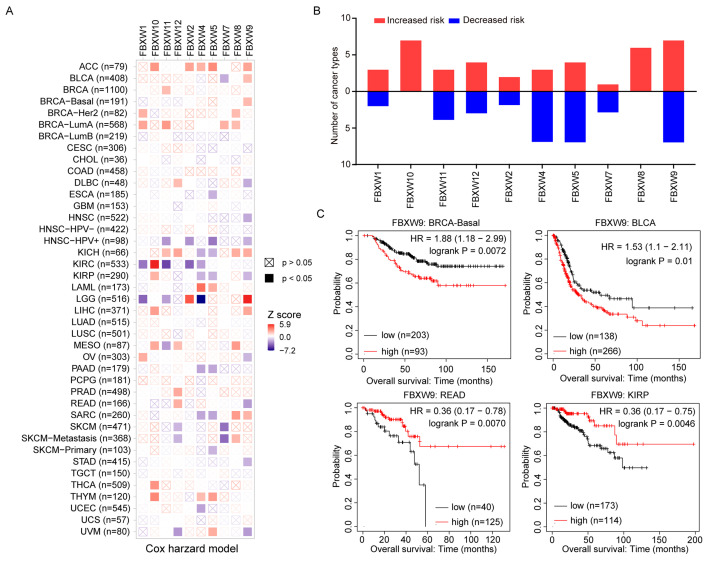
The prognostic value of mRNA levels of FBXWs in multiple cancer types. (**A**) Analysis of the association between mRNA expression of FBXWs with overall survival of patients with 41 cancer types (including subtypes). (**B**) High expression of FBXW9 was associated with poor overall survival of BRCA-Basal and BLCA and good overall survival of READ and KIRP. (**C**) Kaplan-Meier plot of overall survival of patients with BRCA-Basal, BLCA, READ or KIRP.

To study the impact of FBXWs on the prognosis of patients, we collected overall survival data of patients with various cancer types and evaluated the correlation between the expression of FBXWs and prognosis. Based on the TIMER 2.0 database, several cancer types were further divided into subtypes. For example, invasive breast cancer was divided into Basal, Luminal A, Luminal B, and Her2, which gave rise to 41 cancer types. All mRNAs of FBXWs were associated with altered prognosis of cancers of at least four types (Figure 2A,B). To our interest, the expression of FBXW9 was associated with prognosis alternation of many cancer types (14/41), followed by FBXW5 (10/41) and FBXW4 (9/41) (Figure 2A,B). In detail, FBXW9 was favorable (7/41) and hazardous (7/41) to the overall survivals of patients with different cancers, indicating FBXW9 is a double-edged sword in cancers (Figure 2A,B). FBXW4 and FBXW5 were associated with decreased risk of most cancer types examined (7/41). FBXW10, however, was consistently associated with poor prognosis in seven cancer types with significance (Figure 2A,B). Inherently, in BRCA-Basal and BLCA, high expression of FBXW9 predicted poor overall survival with hazard ratios (HR) of 1.88 and 1.53, respectively (Figure 2C). In contrast, high expression of FBXW9 was associated with good prognosis in rectum adenocarcinoma (READ) (HR = 0.36) and kidney renal papillary cell carcinoma (KIRP) (HR = 0.36) (Figure 2C). Overall, these results indicate the important and complicated roles of FBXWs in different cancers. 

### 2.3. Correlation of FBXW Expression and Immune Infiltration across Multiple Cancer Types

**Figure 3:**
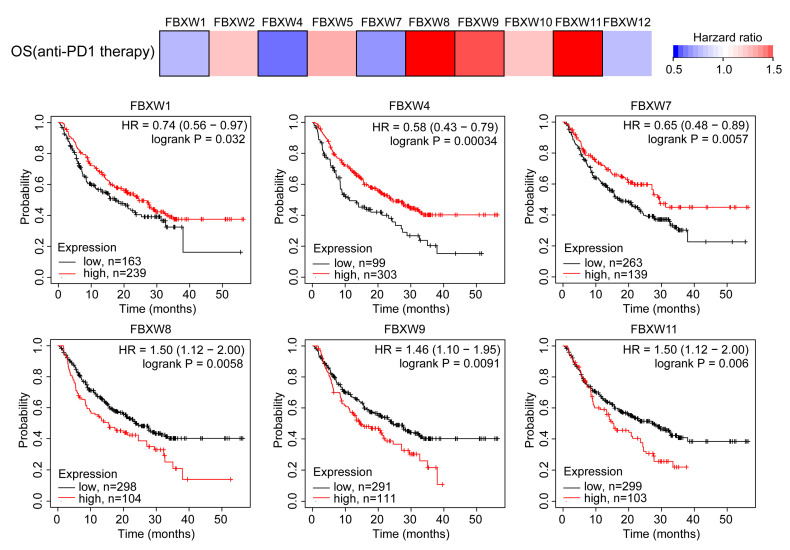
Pan-cancer analysis of expression of FBXWs, tumor microenvironment, and the overall survival of patients receiving anti-PD1 therapy.

**Figure 4:**
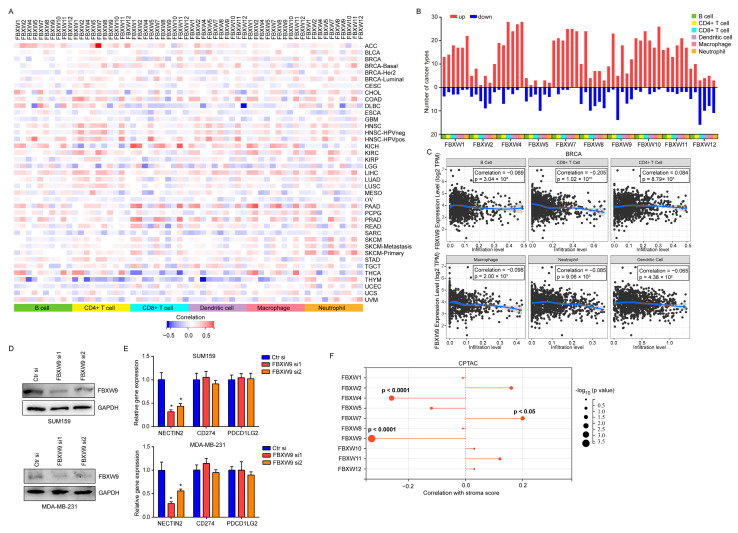
Pan-cancer analysis of expression of FBXWs and tumor microenvironment. (**A**) Heatmap of the association between expression of FBXWs with immune cell infiltrates in multiple cancer types. (**B**) Summary of significant correlations between expression of FBXWs and immune cell infiltrates. (**C**) Correlations between FBXW9 expression and infiltration levels of B cells, CD4+ T cells, CD8+ T cells, dendritic cells, macrophages, and neutrophils were examined in BRCA. (**D**) Western blotting analysis of FBXW9 protein expression in SUM159 and MDA-MB-231 cells transfected with Ctr si, FBXW9 si1, or FBXW9 si2. (**E**) qPCR detection of NECTIN2, CD274, and PDCD1LG2 expression in SUM159 and MDA-MB-231 cells transfected with Ctr si, FBXW9 si1, or FBXW9 si2. (**F**) The association between mRNA expression of FBXWs and stroma score was explored in the CPTAC dataset. *, *p* < 0.05.

Recent studies have shown that several proteins can regulate the function of immune cells to support or suppress cancer progression [16]. We, therefore, analyzed the prognostic value of FBXWs in patients receiving anti-PD1 therapy. In the pan-cancer cohort, high expression of FBXW1, FBXW4, and FBXW7 was strongly associated with prolonged overall survival time in these patients, while FBXW8, FBXW9, and FBXW11 showed the opposite effect (Figure 3). Next, we evaluated the correlation between expression of FBXWs with immune cells in the tumor microenvironment. Overall, FBXWs were positively correlated with the infiltration of all types of immune cells (Figure 4A,B). Specifically, FBXW2, FBXW5, and FBXW12 were mainly negatively associated with the infiltration of immune cells, while FBXW1, FBXW4, FBXW7, and FBXW11 were dominantly positively correlated with the infiltration of immune cells (Figure 4A,B). The impact of FBXW8 and FBXW9 on immune cells was dependent on the cancer context and type of immune cells (Figure 4B,C). For example, in BRCA, FBXW9 was significantly negatively correlated with B cells, CD8+ T cells, macrophages, neutrophils, and dendritic cells while positively correlated with CD4+ T cells (Figure 4C). To investigate the immunosuppressors relevant to FBXW9, the expression of several known immunosuppressors (NECTIN2, CD274, PDCD1LG2) was detected in breast cancer cells [17]. In SUM159 and MDA-MB-231 cells, we showed that the knockdown of FBXW9 decreased NECTIN2 mRNA levels but not CD274 or PDCD1LG2 (Figure 4D,E). We also searched for a correlation between mRNA levels of FBXWs and stroma scores in breast cancer using the CPTAC dataset. mRNA levels of FBXW9 and FBXW4 were negatively associated with stroma score (*p* < 0.001) in breast cancer (Figure 4F). The data implied that FBXWs might be involved in the response to immunotherapy via alteration of the tumor microenvironment. 

### 2.4. Analysis of Correlation between FBXW Expression and Stemness Score

**Figure 5:**
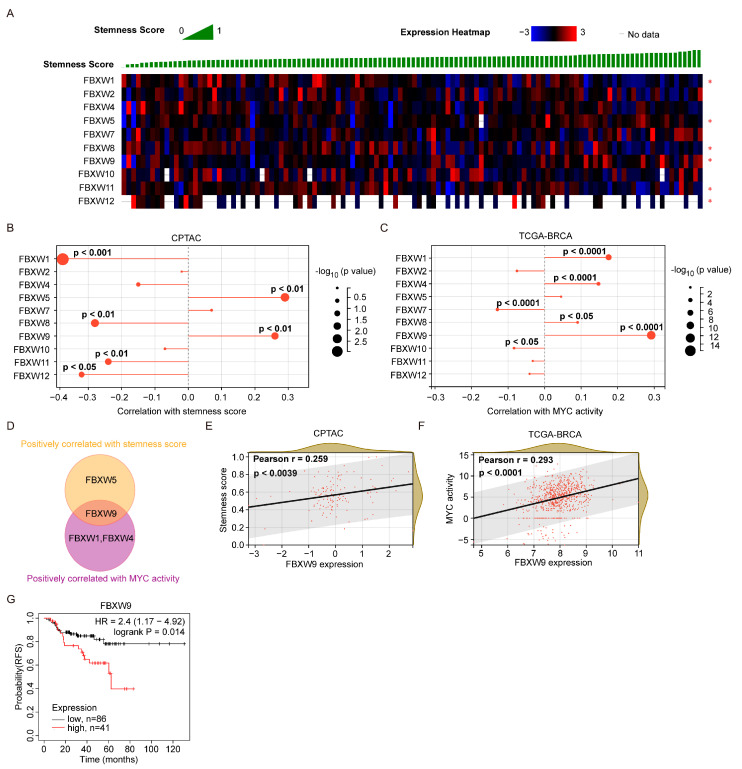
Analysis of the association between expression of FBXWs and stemness in breast cancer. (**A**,**B**) The association between the expression of FBXWs and stemness score was explored in the CPTAC dataset. (**C**) The association between the expression of FBXWs and MYC activity was explored in the TCGA-BRCA dataset. (**D**) Venn diagram analysis of FBXWs associated with stemness score and MYC activity. (**E**,**F**) The correlation between FBXW9 expression and stemness score (**E**) and MYC activity (**F**). (**G**) Kaplan–Meier analysis of the association between FBXW9 expression and relapse-free survival (RFS) of patients with breast cancer.

Cancer stem cells are a small group of cells mediating initiation and drug resistance in cancer cells [18]. To define the association between FBXWs and the stemness of cancers, we collected the machine learning-based stemness score of each sample in the CPTAC dataset. We found that four FBXWs (FBXW-1, 8, 11, and 12) were negatively correlated with stemness score while two FBXWs (FBXW-4 and 9) were positively correlated with stemness score (Figure 5A,B). Since MYC activity was one of the most well-characterized contributors to the stem cell trait in cancer cells [19], we acquired the MYC activity score based on the RABIT transcription factor regulatory impact of each sample from the TCGA-BRCA dataset. Different from the stemness score, four FBXWs (FBXW1, 4, 8, and 9) were positively correlated with the MYC activity while two FBXWs (FBXW7 and 10) were negatively correlated with the MYC activity (Figure 5C). After overlap, no FBXW was both negatively correlated with the stemness score and the MYC activity. Inherently, FBXW9 was positively correlated with both the stemness score and the MYC activity (Figure 5D). In detail, the association between FBXW9 expression and stemness score, and FBXW9 expression and MYC activity were plotted (Figure 5E,F). Due to the critical role of cancer stem cells in mediating chemotherapy resistance, we next analyzed the association between FBXW9 expression and relapse-free survival (RFS) of patients with breast cancer. As expected, patients with high expression of FBXW9 showed significantly shorter RFS compared with those with low expression of FBXW9 (Figure 5G). These data further showed that FBXW9 was the potential contributor to stemness in cancer cells.

### 2.5. Analysis of Potential Substrates of FBXW9

**Figure 6:**
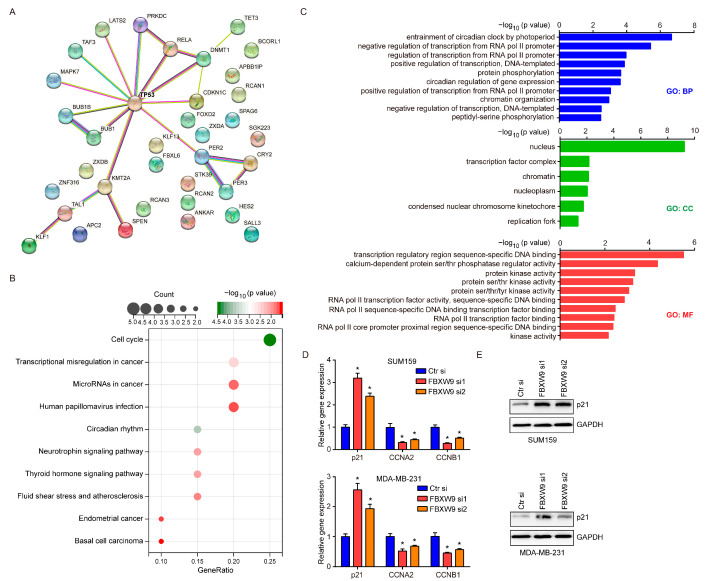
Analysis of potential targets of FBXW9. (**A**) The potential substrates of FBXW9 were predicted using Ubibrowser, and the interaction between these proteins was analyzed using the STRING database. (**B**) KEGG analysis of potential targets of FBXW9 substrates. (**C**) GO analysis of potential targets of FBXW9 substrates. (**D**) qPCR detection of mRNA expression of p21, CCNA2, and CCNB1 in breast cancer cells transfected with Ctr si, FBXW9 si1, or FBXW9 si2. (**E**) Western blotting detection of p21 protein expression in breast cancer cells transfected with Ctr si, FBXW9 si1, or FBXW9 si2. *, *p* < 0.05.

As an E3 ligase, FBXW9 might exert its function by mediating the ubiquitination of substrate proteins. To explore the targets of FBXW9, we used Ubibrowser to perform structure-based predictions. A total of 36 proteins were predicted as potential substrates of FBXW9, and TP53 was the hub gene (Figure 6A). Kyoto Encyclopedia of Genes and Genomes (KEGG) analysis indicated that these proteins were involved in cancer-related processes including cell cycle and transcriptional misregulation in cancer. To our interest, the involvement of basal cell carcinoma (Figure 6B) was consistent with its potential role in basal breast cancer as aforementioned. Gene ontology (GO) analysis further showed that these proteins were mainly localized in the nucleus and were involved in transcription and kinase activities (Figure 6C). As TP53 was the hub gene of putative substrates, we next detected the mRNA expression of p21, a well-characterized target of the TP53 pathway involved in cell cycle arrest [20], and two cell cycle drivers (CCNA2, CCNB1) in breast cancer cells. Indeed, the knockdown of FBXW9 increased p21 mRNA expression while mRNA levels of CCNA2 and CCNB1 were decreased in breast cancer cells (Figure 6D). Moreover, we confirmed the protein expression of p21 was increased in breast cancer cells upon FBXW9 silencing (Figure 6E). The data implied a TP53-regulated cell cycle progression was critical for the function of FBXW9, at least in breast cancer cells. 

### 2.6. Establishment of Signaling Network with Co-Expressed Genes of FBXW9

**Figure 7:**
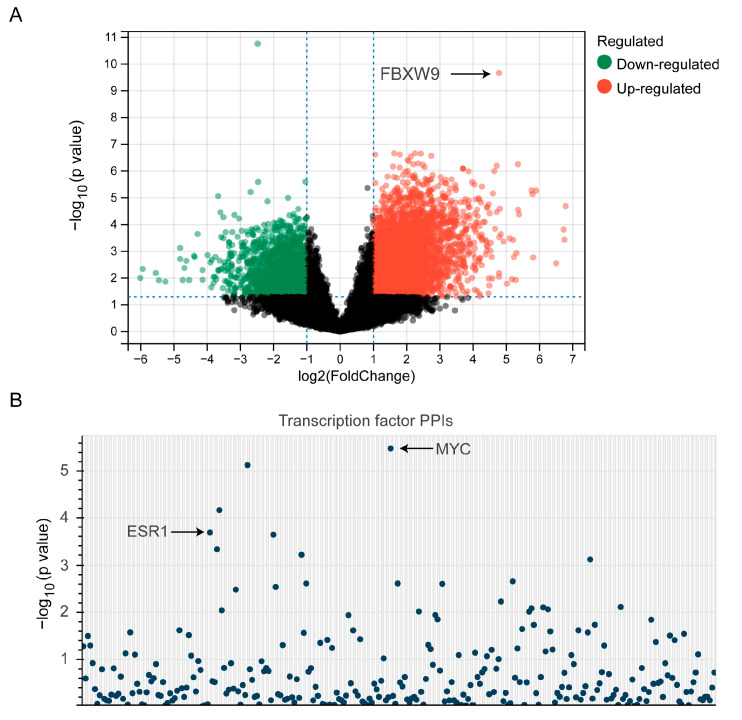
Analysis of genes regulated by FBXW9 in breast cancer. (**A**) The differentially expressed genes between FBXW9 high- and low-expression groups were analyzed. (**B**) The transcription factors regulating the differentially expressed genes were analyzed.

**Figure 8:**
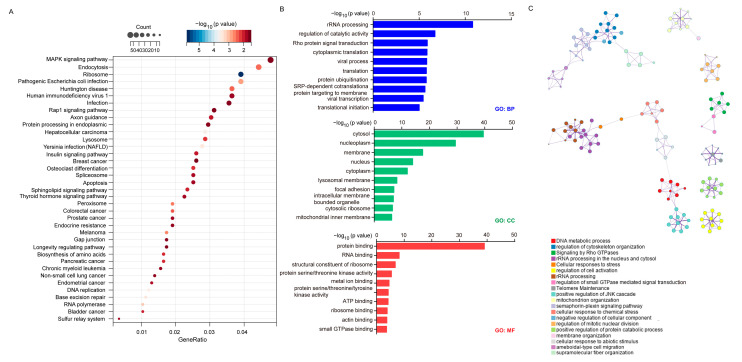
Enrichment analysis of genes regulated by FBXW9 in breast cancer. (**A**) KEGG analysis of the differentially expressed genes. (**B**) GO analysis of the differentially expressed genes. (**C**) Gene enrichment analysis was performed on the differentially expressed genes using Metascape.

To explore the signaling network regulated by FBXW9, we ranked cases in CPTAC according to the protein expression of FBXW9 and picked 10 cases with the highest expression of FBXW9 and 10 cases with the lowest expression of FBXW9. DESeq2 was applied to discover the differentially expressed genes between these two groups. A total of 834 upregulated genes and 2285 downregulated genes were distributed in the FBXW9 high-expression group compared with the counterpart (Figure 7A). Using transcription factor protein–protein interaction networks (PPIs), it was revealed that these genes were targets of well-known oncogenic transcription factors such as ESR1 and MYC (Figure 7B). As MYC is a well-known target of TP53 [21], the transcriptome data implied an FBXW9/TP53/MYC axis in breast cancer. KEGG analysis further indicated that these genes were involved in many oncogenic pathways such as the MAPK signaling pathway and insulin signaling pathway (Figure 8A), which are attributed to several cancer types such as breast cancer, non-small cell lung cancer, colorectal cancer, and hepatocellular carcinoma (Figure 8A). Again, GO analysis indicated that these genes were involved in protein ubiquitination and kinase activities (Figure 8B). Moreover, we used Metascape to establish the signaling network regulated by FBXW9. The plot showed that these differentially expressed genes were involved in several pivotal biological processes for cell migration and proliferation, such as the regulation of cytoskeleton organization, and regulation of mitotic nuclear division (Figure 8C). The signaling network analysis manifested that FBXW9 was potentially involved in cancer progression, particularly in breast cancer.

### 2.7. FBXW9 Silence Inhibited Cell Proliferation and Cell Cycle Progression in Breast Cancer Cells

**Figure 9:**
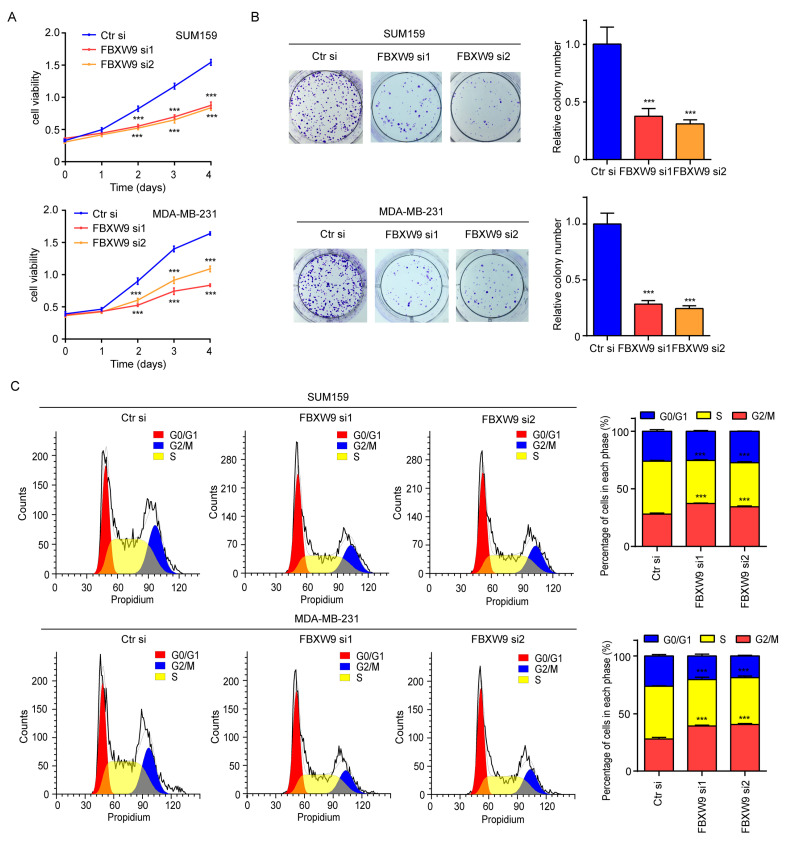
Downregulation of FBXW9 inhibited cell proliferation and cell cycle progression in breast cancer cells. (**A**) The CCK8 analysis was performed on SUM159 cells and MDA-MB-231 with transfection of Ctr si, FBXW9 si1, or FBXW9 si2. (**B**) The colony-forming assay was performed on SUM159 and MDA-MB-231 cells with transfection of Ctr si, FBXW9 si1, or FBXW9 si2. (**C**) Flow cytometry analysis of cell cycle distribution of SUM159 and MDA-MB-231 cells with transfection of Ctr si, FBXW9 si1, or FBXW9 si2. ***, *p* < 0.001.

As evidenced by the bioinformatic data, FBXW9 might contribute to cancer progression including breast cancer. Cell proliferation and colony-forming assays showed that FBXW9 silence notably inhibited cell proliferation (Figure 9A) and decreased the number of cell colonies (Figure 9B) in SUM159 and MDA-MB-231 cells. Flow cytometry analysis further revealed that FBXW9 silence increased the proportion of cells accumulated in the G0/G1 phase, and decreased the proportion of cells in the S phase, indicating alteration of cell cycle distribution (Figure 9C). These data partly confirmed the oncogenic role of FBXW9 in breast cancer.

## 3. Discussion

An imbalance of protein degradation and synthesis leads to the accumulation of oncoproteins or loss of tumor-suppressive proteins, resulting in the initiation and progression of cancers [22]. As a subfamily of E3 ligases, all members of FBXWs contain an F-box motif for forming the SCF complex and several WD40 motifs for interaction with substrates [23]. In addition to these two critical domains, FBXW1 and FBXW11 also contain a D domain for dimerization [24]. Compelling evidence suggested that FBXW1 and FBXW2 were involved in cancer development [25,26]. However, the potential roles of several FBXWs remain elusive. In the present study, we comprehensively analyzed FBXWs in a variety of cancer types. Consistent with the well-characterized anti-tumor function of FBXW4 [27], our analysis also showed that the expression of FBXW4 was decreased in several cancer types, and was associated with a good prognosis of patients. To provide novel insights, the current analysis also revealed prevalent upregulation of FBXW9 and downregulation of FBXW11 in multiple cancer types. Compared with other members, the expression of FBXW9 was associated with overall survival in more cancer types. These data suggested that our analysis could provide profiled information for the expression and prognostic roles of FBXWs in cancers, and implied certain members of FBXWs such as FBXW9 to be potential biomarkers in human cancers. 

Interestingly, the current pan-cancer analysis also provided novel findings uncovering the upregulation of FBXW9 in the majority of cancer types examined, as well as the downregulation of FBXW11 in multiple cancer types. 

FBXW9 is located in 19p13.13 with 10 exons. A previous study showed that FBXW9 was involved in synaptic transmission in C. elegans with an unknown mechanism [28]. Unlike other members of FBXWs, the function and substrates of FBXW9 have not been studied in human diseases yet. According to the protein sequence and structure, we predicted several targets of FBXW9. These putative targets were relevant to TP53 signaling and involved in cell cycle processes, such as TP53, LATS2, and CDKN1C. Via analysis of transcriptome data of breast cancer, FBXW9 was related to the activities of several cell cycle regulators including MYC and ESR1. Interestingly, MYC activity was also regulated by TP53 [21]. In breast cancer cells, we confirmed that the knockdown of FBXW9 inhibited cell proliferation and induced the arrest of cell cycle progression. 

Members of FBXWs showed distinct roles in the prediction of prognosis in patients receiving anti-PD1 therapy, suggesting FBXWs’ different functions in the tumor microenvironment. The percentage of immune cells and non-immune stroma cells infiltrated into tumors could reflect the sensitivity of tumor cells to immune therapy [29]. Generally, infiltration of T cells favors the survival of patients, while stroma cells, in contrast, are linked to poor prognosis of patients [17]. In the Clinical Proteomic Tumor Analysis Consortium (CPTAC) dataset, FBXW4, 7, and 9 were associated with stroma infiltration. Of them, FBXW4 and 9 were strongly negatively correlated with stroma infiltration. For immune cells, FBXWs could be divided into three groups according to their correlation with the infiltration of immune cells. Consistent with the pro-survival role in patients receiving anti-PD1 therapy, FBXW1, FBXW4, and FBXW7 were mainly positively correlated with the infiltration. FBXW9, in contrast, was associated with a poor prognosis in patients receiving anti-PD1 therapy. We experimentally found that FBXW9 repressed the expression of NECTIN2, a regulator of the tumor microenvironment [17], in breast cancer cells.

Cancer stem cells are pivotal for cancer initiation and drug resistance [30]. According to data from breast cancer, it was found that most FBXWs were negatively correlated with stemness except FBXW5 and 9, which were positively correlated with cancer stemness. Consistent with our findings, FBXW1 was verified as an E3 ligase mediating TAZ/YAP degradation, and, thus, contributed to the initiation property and drug resistance of cancer cells [31,32]. Additionally, FBXW9 was positively correlated with the activity of MYC signaling according to RABIT transcription factor regulatory impact, which is a well-known contributor to cancer stemness [33]. This was further validated by analyzing differentially expressed genes in the FBXW9 high-expression group. The data support a potential role of FBXW9 in breast cancer stem cells that needs further experiments for validation. 

## 4. Materials and Methods

### 4.1. Pan-Cancer FBXW Expression Analysis

The TIMER database (https://cistrome.shinyapps.io/timer/, access date: 10 February 2023) was used to explore the transcript expression of FBXWs in cancers from TCGA datasets. The expression pattern of FBXWs was depicted on a heatmap composed of examined cancer types listed in Abbreviation. Pearson correlation analysis was used to analyze the relation between FBXWs across cancer types. The immunohistochemistry data of FBXW9 and FBXW11 in BLCA, BRCA, CESC, LUAD, and corresponding normal tissues were retrieved from the Human Protein Atlas database (www.proteinatlas.org, access date: 10 January 2023).

### 4.2. Pan-Cancer Analysis of the Association between FBXW Expression and Prognosis

TIMER 2.0 (http://timer.cistrome.org/, access date: 10 February 2023) [34] was used to study the association between FBXW expression and the prognosis of 41 cancer types including subtypes of certain major ones. The exploration module was selected and FBXWs were input into the box. The cut-off was selected as *p* < 0.05.

### 4.3. Pan-Cancer Analysis of FBXW Expression and Cancer Immunology

The correlation between FBXWs’ mRNA expression and stroma score was studied using the cBioportal database (https://www.cbioportal.org/, access date: 10 November 2022) relying on the CPTAC dataset [35]. The correlation between FBXW9 mRNA levels and immune cell infiltration was explored using the TIMER database. The association between the expression of FBXWs and patients receiving anti-PD1 therapy was analyzed through a Kaplan–Meier plot (http://kmplot.com/analysis/index.php?p=service&cancer=immunotherapy, access date: 10 February 2023). 

### 4.4. Analysis of FBXWs’ mRNA Expression and Stemness of Breast Cancer

The cBioportal database was used to acquire the stemness score and mRNA expression of FBXWs from each case in CPTAC. The XENA database (https://xenabrowser.net/, access date: 10 November 2022) was used to study the association between MYC activity and mRNA expression of FBXWs in the TCGA-BRCA dataset. The MYC activity was calculated using the RABIT transcription factor regulatory impact [36]. 

### 4.5. Prediction of Substrates of FBXW9

The UbiBrowser 2.0 software (http://ubibrowser.bio-it.cn/ubibrowser_v3/, access date: 5 November 2022) [37] was chosen to predict substrates of FBXW9. The protein interaction was analyzed using the STRING database (https://cn.string-db.org/, access date: 5 November 2022). The predicted substrates were further analyzed through KEGG and GO analyses using the DAVID database (https://david.ncifcrf.gov/summary.jsp, access date: 5 November 2022).

### 4.6. Pathway Enrichment Analysis of FBXW9-Correlated Genes in Breast Cancer

We downloaded proteomic data on breast cancer from CPTAC. The cases were ranked according to FBXW9 protein expression. After that, we selected 10 cases with the highest FBXW9 expression (FBXW9 high-expression group) and 10 cases with the lowest FBXW9 expression (FBXW9 low-expression group). We next downloaded transcriptome data of 20 cases including the FBXW9 high- and low-expression groups. The DEseq2 package was used to analyze differentially expressed genes between the two groups. The cut-off was *p* < 0.05 and |Fold change| > 1. The differentially expressed genes were again sent to KEGG analysis and GO analysis using the DAVID database. They were also used to build a signaling network with Metascape (https://metascape.org/gp/index.html#/main/step1, access date: 5 January 2023). 

### 4.7. Cell Culture

Triple-negative breast cancer cell lines SUM159 and MDA-MB-231 were purchased from ATCC and maintained in DMEM with 10% FBS (Hyclone, UT, USA). The cells were cultured in a humid incubator (37 °C, 5% CO_2_). 

### 4.8. siRNA-Mediated Gene Silencing

Specific siRNAs targeting FBXW9 and control siRNA were purchased from GenePharma (Shanghai, China). The siRNAs were transfected into SUM159 and MDA-MB-231 cells using LipoFectamine RNAiMax (Invitrogen, CA, USA) reagent according to the manufacturers’ protocol. 

### 4.9. Cell Proliferation and Colony Forming Assays

The proliferation ability of cells was determined with a CCK-8 kit (Dojindo, Tokyo, Japan) following the manufacturer’s protocol. For the colony-forming assay, cells were transfected with siRNAs and then plated in 6-well plates and cultured for 7 days. The cells were stained with crystal violet.

### 4.10. Cell Cycle Analysis

Cell cycle analysis was conducted as previously described [38]. In short, Cells were fixed in 70% ethanol for 1 h at 4 °C, and then were treated with RNase and stained with PI for 30 min at room temperature. The cells were then subjected to flow cytometry analysis using ModFit software (Version 3.1).

### 4.11. Human Tissues Collection

The breast tumors and matched normal tissues were collected from surgery from 25 patients in the Affiliated Hospital of Yangzhou University from 2021 to 2022. Written informed consent was acquired from all participants and the study was under the supervision of the Ethic Committee of the Affiliated Hospital of Yangzhou University (YXYLL-2021-07). No therapy was received before the surgery. The samples were immediately subjected to RNA extraction using TRIzol reagent.

### 4.12. RT-qPCR

RNA was extracted from cells with Trizol reagent (Invitrogen). The RNA was reverse-transcribed with HiScript Reverse Transcriptase (Vazyme, Nanjing, China) and RT-qPCR was performed with ChamQ Universal SYRB qPCR Master Mix (Vazyme). The primer sequences were as follows: FBXW9-F:5′-TAGGGCGGTGCGATGATTC-3′; FBXW9-R:5′-CGGATTTTGGCGGACTGAGA-3′; p21-F: 5′-TGTCCGTCAGAACCCATGC-3′; p21-R:5′-AAAGTCGAAGTTCCATCGCTC-3′; CCNA2-F:5′-CGCTGGCGGTACTGAAGTC-3′; CCNA2-R: 5′-GAGGAACGGTGACATGCTCAT-3′; CCNB1-F:5′-AATAAGGCGAAGATCAACATGGC-3′; CCNB1-R:5′-TTTGTTACCAATGTCCCCAAGAG-3′; NECTIN2-F:5′-GGATGTGCGAGTTCAAGTGCT-3′; NECTIN2-R:5′-TGGGACCCATCTTAGGGTGG-3′; CD274-F:5′-TGGCATTTGCTGAACGCATTT-3′; CD274-R:5′-TGCAGCCAGGTCTAATTGTTTT-3′; PDCD1LG2-F:5′-ATTGCAGCTTCACCAGATAGC-3′; PDCD1LG2-R:5′-AAAGTTGCATTCCAGGGTCAC-3′; 18S-F:5′-GTAACCCGTTGAACCCCATT-3′;18S-R:5′-CCATCCAATCGGTAGTAGCG-3′. 

### 4.13. Western Blotting

Protein was extracted from cells using RIPA lysis buffer (Beyotime, Shanghai, China). Lysates were loaded on an SDS-PAGE gel. After electrophoresis, proteins were transferred to a PVDF membrane. The membrane was blocked with 5% non-fat milk and then incubated with the primary and secondary antibodies sequentially. The blots were developed with Enhanced ECL buffer (Beyotime). The information of antibodies was as follows: FBXW9 (Novus Biologicals, CO, USA), p21 (Proteintech, IL, USA), GAPDH (Proteintech), HRP-conjugated anti-mouse (Proteintech), HRP-conjugated anti-rabbit (Proteintech).

### 4.14. Statistical Analysis

The data were analyzed and graphed using GraphPad Prism 6 and presented as the mean ± SD of three repeats. Two-group comparison was performed with Student’s *t*-test. A *p* < 0.05 was considered statistically significant. 

## 5. Conclusions

In conclusion, the comprehensive analysis of FBXWs suggested that several novel E3 ligases might be involved in cancer progression. Importantly, the data strongly suggested a critical role of FBXW9 in several cancer types. FBXW9 might govern cell cycle, stemness, and immune infiltration in cancers. In basal breast cancer, FBXW9 promoted cell cycle progression to facilitate cell growth. However, further studies to explore the molecular mechanisms of FBXWs are needed in the future.

## Data Availability

All data generated or analyzed are included in the current manuscript and they are available from the corresponding author upon reasonable request.

## Supplementary Materials

The following supporting information can be downloaded at: https://www.mdpi.com/article/10.3390/ijms24065262/s1.

## Abbreviations

ACC	Adrenocortical carcinoma
BLCA	Bladder urothelial carcinoma
BRCA	Breast invasive carcinoma
CESC	Cervical squamous cell carcinoma and endocervical adenocarcinoma
CHOL	Cholangiocarcinoma
COAD	Colon adenocarcinoma
DLBC	Lymphoid neoplasm diffuse large B-cell lymphoma
ESCA	Esophageal carcinoma
GBM	Glioblastoma multiforme
HNSC	Head and neck squamous cell carcinoma
KICH	Kidney chromophobe
KIRC	Kidney renal clear cell carcinoma
KIRP	Kidney renal papillary cell carcinoma
LAML	Acute myeloid leukemia
LGG	Brain lower-grade glioma
LIHC	Liver hepatocellular carcinoma
LUAD	Lung adenocarcinoma
LUSC	Lung squamous cell carcinoma
MESO	Mesothelioma
OV	Ovarian serous cystadenocarcinoma
PAAD	Pancreatic adenocarcinoma
PCPG	Pheochromocytoma and paraganglioma
PRAD	Prostate adenocarcinoma
READ	Rectum adenocarcinoma
SARC	Sarcoma
SKCM	Skin cutaneous melanoma
STAD	Stomach adenocarcinoma
TGCT	Testicular germ cell tumors
THCA	Thyroid carcinoma
THYM	Thymoma
UCEC	Uterine corpus endometrial carcinoma
UCS	Uterine carcinosarcoma
UVM	Uveal melanoma
