# Supplementary figures and images for: A Comprehensive Analysis Revealing FBXW9 as a Potential Prognostic and Immunological Biomarker in Breast Cancer
Source: Int J Mol Sci. 2023 Mar 9;24(6):5262. doi: 10.3390/ijms24065262 (PMC10049633; doi:10.3390/ijms24065262)

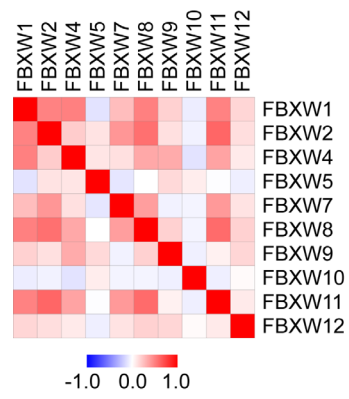

**Figure S1.** The correlations between members of FBXW were analyzed in cancers.

Supplement: Supplementary file 1 [file ijms-24-05262-s001.zip › ijms-2197163-supplementary.pdf]
